# Effect of menopausal hormone therapy on methylation levels in early and late postmenopausal women

**DOI:** 10.1186/s13148-022-01311-w

**Published:** 2022-07-18

**Authors:** James R. Hilser, Jaana A. Hartiala, Intira Sriprasert, Naoko Kono, Zhiheng Cai, Roksana Karim, Joseph DeYoung, Wendy J. Mack, Howard N. Hodis, Hooman Allayee

**Affiliations:** 1 Departments of Population and Public Health Sciences, Keck School of Medicine, University of Southern California, 2250 Alcazar Street, CSC202, Los Angeles, CA 90033 USA; 2 Departments of Biochemistry and Molecular Medicine, Keck School of Medicine, University of Southern California, Los Angeles, CA 90033 USA; 3 Departments of Obstetrics and Gynecology, Keck School of Medicine, University of Southern California, Los Angeles, CA 90033 USA; 4 Departments of Medicine, Keck School of Medicine, University of Southern California, Los Angeles, CA 90033 USA; 5 Atherosclerosis Research Unit, Keck School of Medicine, University of Southern California, Los Angeles, CA 90033 USA; 6 Department of Psychiatry and Biobehavioral Sciences, Center for Neurobehavioral Genetics, Semel Institute for Neuroscience and Human Behavior, David Geffen School of Medicine of UCLA, Los Angeles, CA 90095 USA

**Keywords:** Methylation, Hormone therapy, Menopause, Atherosclerosis

## Abstract

**Background:**

Cardiovascular disease (CVD) remains the leading cause of death among postmenopausal women but standard primary prevention strategies in women are not as effective as in men. By comparison, the Early versus Late Intervention Trial with Estradiol (ELITE) study demonstrated that hormone therapy (HT) was associated with significant reduction in atherosclerosis progression in women who were within six years of menopause compared to those who were 10 or more years from menopause. These findings are consistent with other studies showing significant reductions in all-cause mortality and CVD with HT, particularly when initiated in women younger than 60 years of age or within 10 years since menopause. To explore the biological mechanisms underlying the age-related atheroprotective effects of HT, we investigated changes in methylation of blood cells of postmenopausal women who participated in ELITE.

**Results:**

We first validated the epigenetic data generated from blood leukocytes of ELITE participants by replicating previously known associations between smoking and methylation levels at previously identified CpG sites, such as cg05575921 at the *AHRR* locus. An epigenome-wide association study (EWAS) evaluating changes in methylation through interactions with time-since-menopause and HT revealed two significantly associated CpG sites on chromosomes 12 (cg19552895; *p* = 1.1 × 10^–9^) and 19 (cg18515510; *p* = 2.4 × 10^–8^). Specifically, HT resulted in modest, but significant, increases in methylation levels at both CpGs but only in women who were 10 or more years since menopause and randomized to HT. Changes in carotid artery intima-media thickness (CIMT) from baseline to 36 months after HT were not significantly correlated with changes in methylation levels at either cg19552895 or cg18515510. Evaluation of other previously identified CpG sites at which methylation levels in either blood or vascular tissue were associated with atherosclerosis also did not reveal any differences in methylation as a function of HT and time-since-menopause or with changes in CIMT.

**Conclusions:**

We identified specific methylation differences in blood in response to HT among women who were 10 or more years since menopause. The functional consequence of these change with respect to atherosclerosis progression and protective effects of HT remains to be determined and will require additional studies.

## Background

Cardiovascular disease (CVD) remains the leading cause of death among postmenopausal women [1]. Although standard primary prevention with statins, aspirin, and ACE inhibitors significantly reduces CVD risk in men, the cardioprotective effects in women are less certain with no reduction in all-cause mortality [2–8]. By comparison, hormone therapy (HT) has been shown to reduce all-cause mortality and CVD in primary prevention, when initiated in women younger than 60 years or who are less than 10 years since menopause [9–11]. These meta-analyses of randomized controlled trials are consistent with results from the randomized, double-blinded, placebo-controlled Early versus Late Intervention Trial with Estradiol (ELITE) that specifically tested the effect of HT on subclinical atherosclerosis as a function of time-since-menopause [12]. ELITE confirmed the HT timing hypothesis by demonstrating that, compared with placebo, carotid artery intima-media thickness (CIMT) progression was significantly lowered by HT when initiated within 6 years of menopause but had no effect on CIMT progression when initiated 10 years or more after menopause [12]. Thus, results of ELITE clearly indicated a sex-specific and age-related opportunity for reducing CVD and all-cause mortality trends in women.

Despite the benefits of HT shown in ELITE and other studies, biological mechanisms underlying the age-related atheroprotective effects of HT remain unknown and cannot be completely explained by effects on known risk factors. Possible explanations for these observations at the molecular level may be related to epigenetic modification, expression, and/or signaling of estrogen receptors (ESRs) in atherosclerosis-related tissues as a function of aging and/or time-since-menopause. For example, in women, estradiol has been shown to upregulate *ESR1* and *ESR2* mRNA levels in leukocytes, such as macrophages and neutrophils [13, 14], and age-related increases in methylation of CpG islands in the promoters of both *ESR1* and *ESR2* has been observed in atherosclerotic and normal vascular tissue [15, 16] as well as in proliferating smooth muscle cells that are characteristically found in atherosclerotic lesions [17]. The promoter regions of genes in other atherosclerosis relevant genes, such as the pro-inflammatory enzyme 15-lipoxygenase, have also exhibited significantly decreased methylation in advanced human atherosclerotic lesions compared with fatty streaks, which was accompanied by abundant 15-lipoxygenase mRNA levels [18].

In the present study, we sought to explore the potential molecular mechanisms by which HT decreased subclinical atherosclerosis progression among participants of ELITE. An unbiased epigenome-wide association study (EWAS) was carried out to identify CpG sites at which methylation levels changed in response to HT as function of time-since-menopause. Candidate loci were further evaluated bioinformatically and for association with CIMT progression.

## Results

### Characteristics of the study population

Based on the findings of ELITE [12], we designed a study to maximize the likelihood of identifying differentially methylated CpG sites as a function of time-since-menopause and HT. We selected subset of 48 women from the early/HT with the lowest 36-month rate of CIMT progression and an equivalent number of women from each of the early/placebo, late/placebo, and late/HT groups with the highest CIMT progression for the present analysis. As shown in Table **1**, there were no significant differences in baseline clinical or demographic characteristics across the four study groups apart from expected differences in age, years since menopause, and progression of CIMT after 36 months of HT.

**Table 1:** Clinical characteristics of study participants

Trait (N or %)	Early/Placebo (*n* = 48)	Early/Treatment (*n* = 48)	^a^Late/Placebo (*n* = 42)	Late/Treatment (*n* = 48)	^b^*p*-value
Age	55.5 (5.2)	55.3 (4.7)	65.1 (7.8)	66.7 (8.1)	< 0.0001
Years since menopause	3.3 (3.1)	3.5 (3.5)	14.6 (7.9)	14 (6.7)	< 0.0001
*Ethnicity*					0.68
White Non-Hispanic	34 [71]	39 [81]	30 [71]	33 [44]	
Black Non-Hispanic	8 [17]	2 [4]	4 [45]	6 [13]	
Hispanic	3 [6]	5 [45]	6 [14]	5 [45]	
Asian	3 [6]	2 [4]	2 [46]	4 [8]	
*Smoking*					0.70
Never	24 [50]	28 [58]	24 [57]	33 [44]	
Former	22 [46]	19 [46]	17 [46]	14 [29]	
Current	2 [4]	1 [2]	1 [2]	1 [2]	
Body mass index	27.0 (6.6)	26.1 (5.3)	27.1 (5.5)	26.2 (7.8)	0.90
Total cholesterol, mg/dL	233 (46)	231 (30)	227 (30)	222 (53)	0.64
LDL, mg/dL	144 (41)	147 (37)	137 (42)	133 (52)	0.30
HDL, mg/dL	64 (26)	60 (20)	63 (29)	59 (22.5)	0.73
SBP, mmHg	120 (21)	117 [15]	121 (19)	122 [13]	0.09
DBP, mmHg	77 (10)	77 (9)	75 (7)	75 [11]	0.47
*Change in free estradiol (pg/mL)	0.015 (0.09)	0.38 (0.45)	0.01 (0.12)	0.35 (0.70)	< 0.0001
*Change in CIMT, mm	0.048 (0.017)	− 0.011 (0.029)	0.057 (0.014)	0.059 (0.014)	< 0.0001

Data are shown as median (IQR) or as n [%]

^*^Change is shown as the difference between baseline and 36 months after treatment (calculated as post–pre)

^a^Six subjects in the late/placebo group whose methylation data did not pass QC steps were excluded from all analyses

^b^P-values for differences between groups were derived from Kruskal–Wallis tests for continuous variables or chi-square tests for dichotomous/categorical traits, respectively

### Association of methylation with smoking

**Fig. 1:**
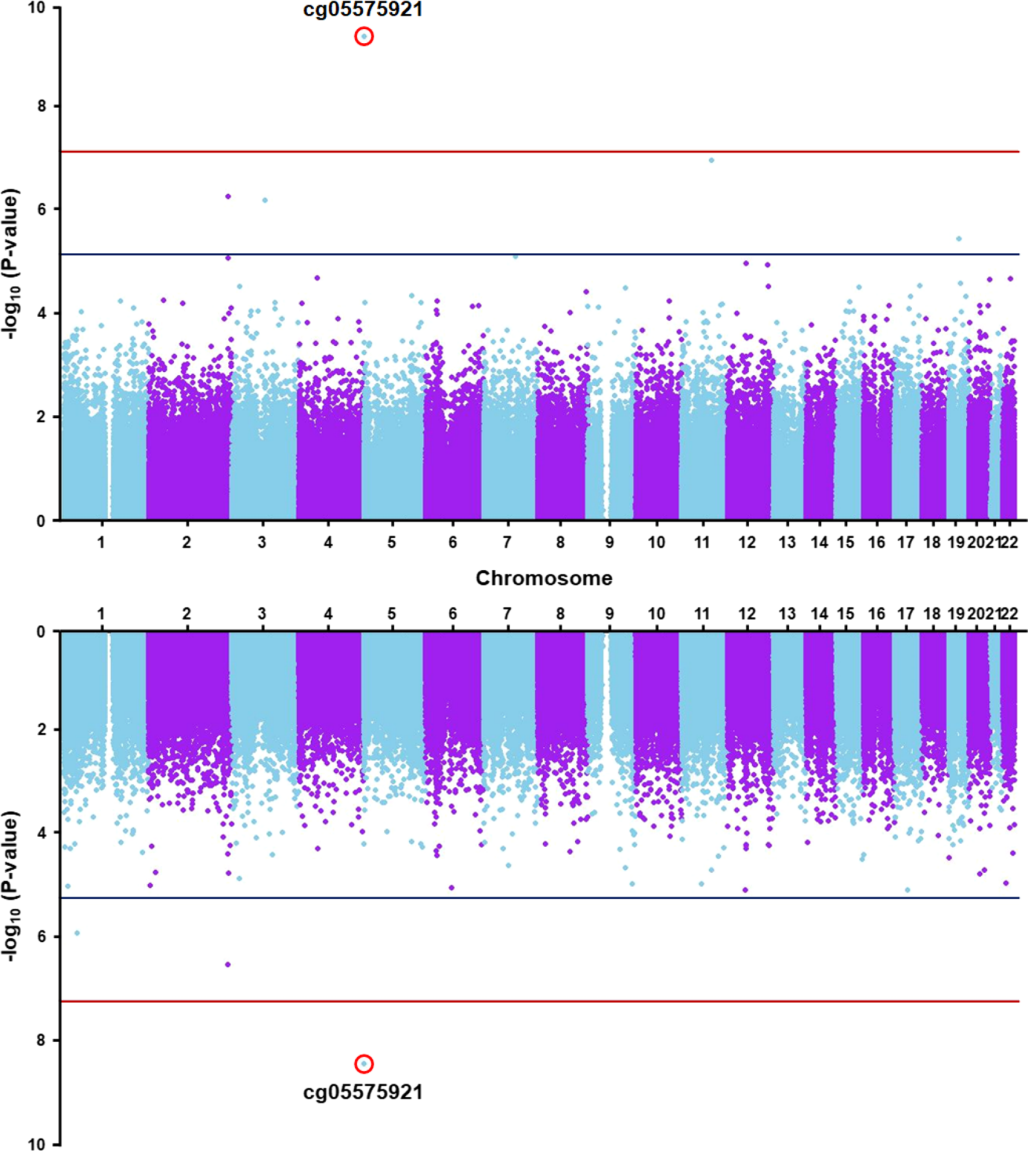
Miami plot of EWAS results for association of methylation levels with smoking. Methylation levels at a CpG site on chromosome 5 (cg05575921) were significantly associated with smoking at baseline (top panel) and 36 months after hormone treatment (bottom panel). Genome-wide methylation was assessed across 748,567 CpG sites and *p*-values for differences between never, former, and current smokers, as determined by linear regression using M-values for methylation with adjustment for age, ethnicity, time-since-menopause, treatment, and estimated blood cell fractions, are plotted as a function of genomic location. The solid red and blue lines indicate the significant (*p* = 6.7 × 10^–8^) and suggestive (*p* = 6.7 × 10^–6^) thresholds for significance, respectively

As an initial step in our analyses, we validated methylation data generated from buffy coat-derived blood leukocytes of all selected ELITE participants by carrying out an EWAS for smoking. We chose this exposure as a representative outcome since previous studies have identified strong and reproducible methylation signals in blood DNA at multiple sites associated with smoking [19]. Consistent with prior studies, comparisons between never, former, and current smokers revealed associations between smoking and methylation levels at both baseline and 36 months post HT with several of the five CpG sites previously reported to be most strongly affected by smoking (Table 2). At these CpGs, smoking was associated with decreased methylation levels, which is directionally consistent with the observed effects of smoking in numerous previous studies [19]. In particular, the association signals at cg05575921 in intron 3 of the aryl hydrocarbon receptor repressor gene (*AHRR*) on chromosome 5 exceeded the Bonferroni-corrected genome-wide significance threshold for testing 748,567 CpGs (*p* = 0.05/748,567 = 6.7 × 10^–8^) (Fig. 1; Table 2). Nominally significant (*p* < 0.05) associations between methylation levels and smoking were also observed at three of the four other selected smoking-associated CpG sites at baseline and 36 months after treatment, although only cg19859270 and cg03636183 are considered significant at a Bonferroni threshold for testing five CpGs (*p* = 0.05/5 = 0.01; Table 2). Thus, these data validate the quality and suitability of the methylation data generated from buffy coats of ELITE participants for EWAS analyses.

**Table 2:** Methylation at previously identified CpGs most strongly affected by smoking

CpG	^a^Chr:pos	Nearest gene	Baseline		36-month visit	
			Effect (SE)	^b^*p*-value	Effect (SE)	^b^*p*-value
cg09935388	1:92947588	*GFI1*	− 0.094 (0.063)	0.14	− 0.063 (0.057)	0.27
cg19859270	3:98251294	*GPR15*	− 0.078 (0.023)	9.8 × 10^–4^	− 0.045 (0.023)	0.047
cg23576855	5:373299	*AHRR*	− 0.24 (0.12)	0.04	− 0.25 (0.12)	0.04
cg05575921	5:373378	*AHRR*	− 0.46 (0.07)	3.9 × 10^–10^	− 0.44 (0.072)	4.0 × 10^–9^
cg03636183	19:17000585	*F2RL3*	− 0.13 (0.033)	2.4 × 10^–4^	− 0.12 (0.030)	1.4 × 10^–4^

Data are shown as the effect of smoking on methylation (M-values) at the baseline visit and 36 months after treatment across all treatment groups

^a^Chromosome and position (base pair) are based on build 37 (hg19) of the human genome reference sequence

^b^*p*-values were derived from linear regression analyses for smoking status (never, former, or current; coded as 0, 1, 2) with adjustment for age, ethnicity, time-since-menopause, randomized treatment, and estimated blood cell fractions

### Differential methylation as function of time-since-menopause and HT

**Fig. 2:**
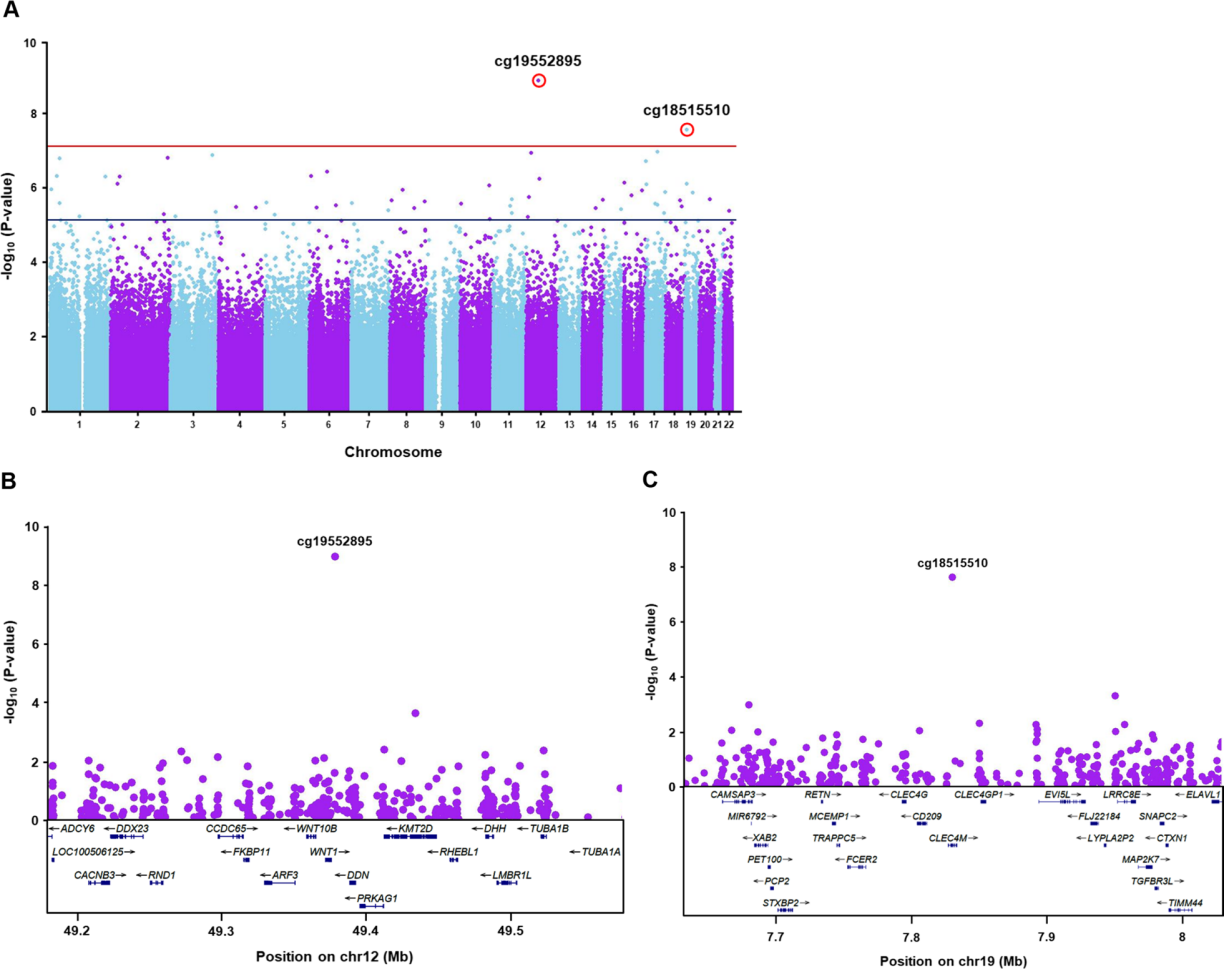
EWAS results for association of methylation levels with time-since-menopause and HT. (**A**) Manhattan plot shows two CpG sites on chromosomes 12 (cg19552895) and 19 (cg18515510) at which the difference in methylation levels between baseline and 36 months after treatment were significantly associated with time-since-menopause and treatment. Genome-wide methylation was assessed across 748,567 CpG sites and interaction P-values between time-since-menopause and HT for changes in methylation (M-values) from baseline to 36 months post treatment, with adjustment for age, ethnicity, and estimated blood cell fractions, are plotted as a function of genomic location. The solid red and blue lines indicate the significant (*p* = 6.7 × 10^–8^) and suggestive (*p* = 6.7 × 10^–6^) thresholds for significance, respectively. Regional plots show 400kb intervals on chromosomes 12 and 19 centered on cg19552895 (**B**) and cg18515510 (**C**), respectively. Genes located within the 400kb intervals are shown in the bottom panels

**Fig. 3:**
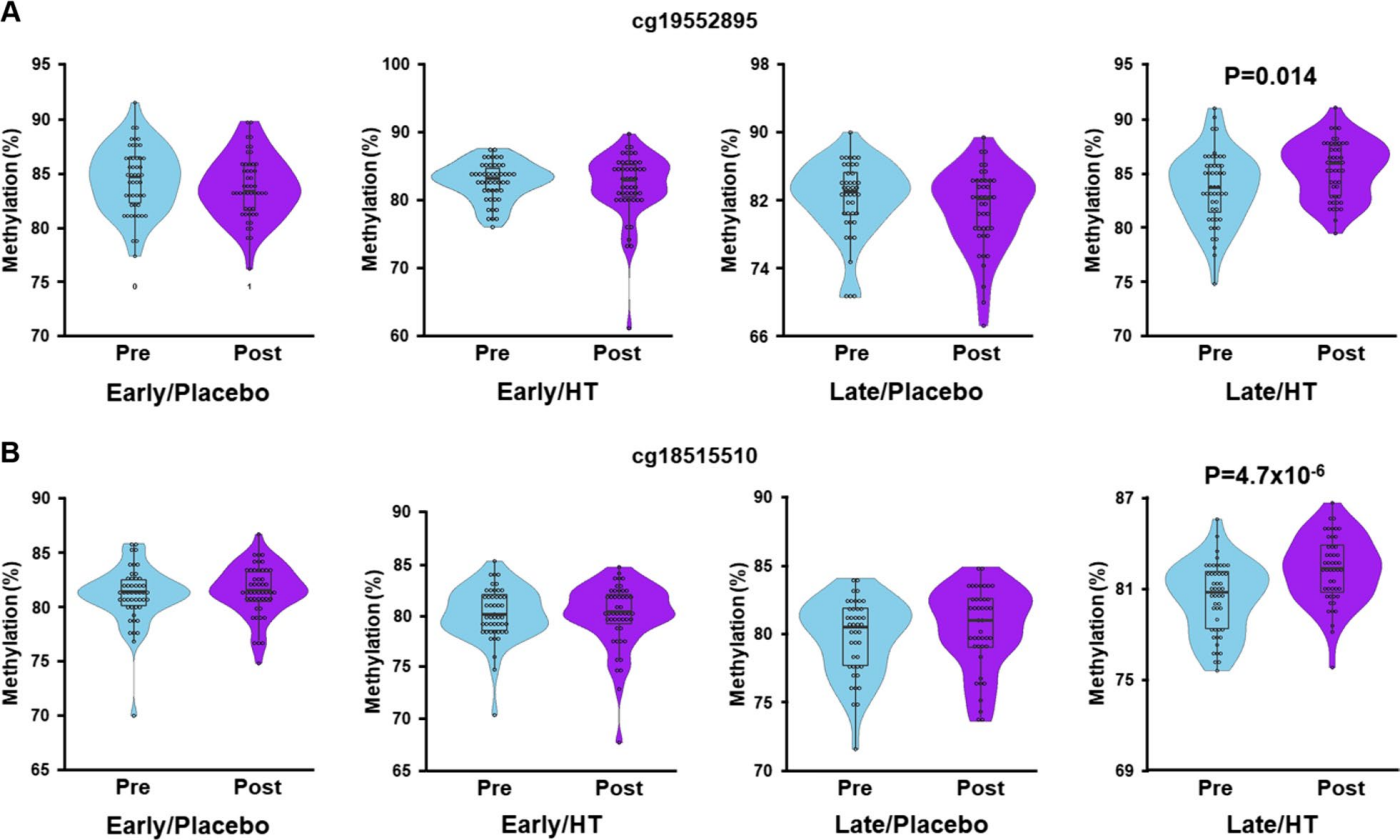
Methylation levels at two significantly associated CpG sites as a function of treatment group. Methylation levels (%) for cg19552895 (**A**) and cg18515510 (**B)** at baseline and 36 months after treatment are shown in the four treatment groups of women. Significant increases in methylation were observed from baseline (pre) to 36 months after treatment (post) for both CpGs in the late (> 6 years from menopause) HT group but not any of the other three groups. P-values are based on linear regression using M-values for methylation, with adjustment for age, ethnicity, and estimated blood cell fractions

We next carried out an EWAS analysis to identify differentially methylated regions of the genome associated with time-since-menopause and HT. Two CpG sites located on chromosomes 12 (cg19552895; p-int = 1.1 × 10^–9^) and 19 (cg18515510; p-int = 2.4 × 10^–8^) yielded genome-wide significant p-values (*p* < 6.7 × 10^–8^) for interaction of time and the time-since-menopause/HT groups, indicating differences in the 36-month changes in methylation over the four groups  (Fig. 2A). Cg19552895 maps to a shelf region downstream of *WNT1* whereas cg18515510 is located in the 3’ UTR of *CLEC4M* (Fig. 2B, C). The interactions between time-since-menopause and HT for methylation differences between baseline and 36 months after treatment at both CpGs were based on a ~ 2% increase after treatment in only the late postmenopausal HT group (Table 3; Fig. 3). In addition to cg19552895 and cg18515510, numerous other CpG sites distributed throughout the genome also yielded suggestive (p-int < 6.7 × 10^–6^) interactions with time-since-menopause and HT (Fig. 2A).

**Table 3:** CpG sites with significant changes in percent methylation as a function of menopause and treatment groups

CpG	^a^Chr:pos	Nearest gene (location)	Early/Placebo (*n* = 48)	Early/Treatment (*n* = 48)	Late/Placebo (*n* = 42)	Late/Treatment (*n* = 48)	^b^P-int
cg19552895	12:49379205	*WNT1* (S_Shelf)	− 0.8	− 0.6	− 1.2	1.7	1.1 × 10^–9^
cg18515510	19:7831896	*CLEC4M* (3' UTR)	0.4	− 0.2	0.6	1.9	2.4 × 10^–8^

Data are shown as the mean change in methylation levels (%) between baseline and 36 months after treatment (calculated as post–pre) for the four treatment groups

^a^Chromosome and position (base pair) are based on build 37 (hg19) of the human genome reference sequence

^b^*p*-values are derived from tests of interaction between time-since-menopause and HT using M-values for methylation with adjustment for age, ethnicity, and estimated blood cell fractions

S_Shelf, southern shelf region that is directly adjacent to a southern shore, which is directly downstream of a CpG island

### Association between differentially methylated CpGs and CIMT progression

**Fig. 4:**
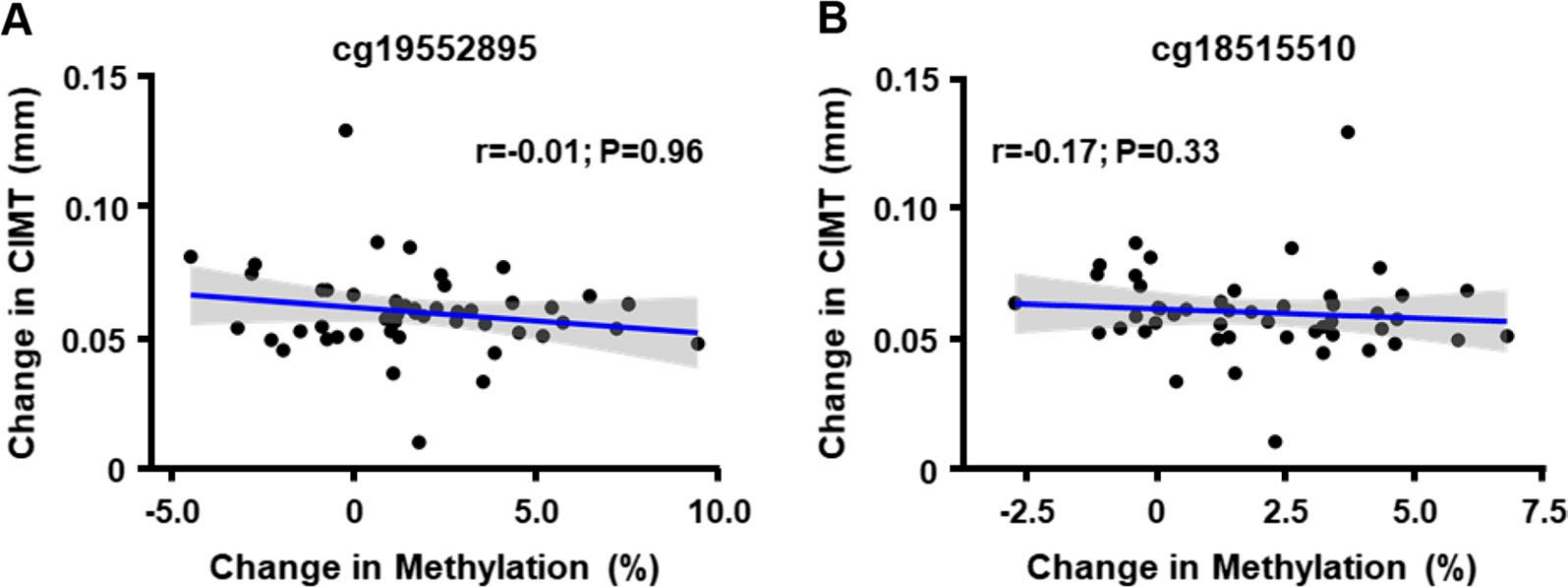
Relationship between changes in methylation levels and subclinical atherosclerosis. The correlation between changes in CIMT and changes in methylation levels (%) of cg19552895 (**A**) and cg18515510 (**B**) from baseline to 36 months after treatment (calculated as post–pre for CIMT and methylation) is shown for women in the late/HT group. *p*-values are based on Spearman correlations using M-values for methylation with adjustment for age and estimated blood cell fractions at baseline and at 36 months after treatment

We next evaluated whether the increased methylation at cg19552895 and cg18515510 as a result of HT was associated with CIMT progression. Among women in the late/HT group, changes in CIMT from baseline to 36 months after HT were not significantly correlated with differences in methylation levels at either CpG (Fig. 4). A similar analysis with changes in free estradiol levels from baseline to 36 months post treatment also did not reveal a relationship with changes in methylation levels at cg19552895 (*r* = − 0.18; *p* = 0.29). Although there was a modest positive correlation with cg18515510 (*r* = 0.36; *p* = 0.033), it would not be considered significant at a Bonferroni-corrected p-value for testing 2 CpGs (*p* = 0.05/2 = 0.025). Lastly, we also evaluated other previously identified CpG sites at which methylation levels in either blood or vascular tissue were associated with atherosclerosis, including those related to smoking [20–22]. However, none of the selected regions exhibited differences in methylation among women in the four treatment groups that would be considered significant for the number of CpG sites tested (Table 4).

**Table 4:** Methylation levels in leukocytes of ELITE participants at CpGs previously associated with atherosclerosis

Tissue	CpG	^a^Chr:pos	Nearest Gene	Early/Placebo (*n* = 48)	Early/Treatment (*n* = 48)	Late/Placebo (*n* = 42)	Late/Treatment (*n* = 48)	^b^P-int
Aortic plaque	cg07608848	2:1,647,185	*PXDN*	0.4	0.1	0.1	0.2	0.43
Blood	cg23079012	2:8,343,662	*LINC00299*	− 0.2	0.3	0.2	0.1	0.66
Blood	cg21566642	2:233,284,613	*ALPI*	− 0.7	0.3	0.1	0.01	0.08
Blood	cg03358636	3:197,473,958	*RUBCN*	0.5	1.3	− 0.4	0.1	0.73
Blood/Carotid plaque	cg12806681	5:368,346	*AHRR*	0.1	0.1	0.3	0.3	0.82
Blood	cg23916896	5:368,756	*AHRR*	1.0	0.9	0.1	0.7	0.16
Blood/Carotid plaque	cg05575921	5:373,378	*AHRR*	− 0.1	− 0.2	0.6	− 0.2	0.63
Blood	cg26703534	5:377,358	*AHRR*	0.5	0.1	− 0.6	− 0.3	0.81
Blood	cg21161138	5:399,312	*AHRR*	0.6	0.1	− 0.5	− 0.1	0.97
Aortic plaque	cg23979631	7:27,142,427	*HOXA2*	0.2	1.0	0.0	0.0	0.36
Aortic plaque	cg19816811	7:27,188,364	*HOXA6*	1.0	0.7	0.1	− 0.5	0.28
Aortic plaque	cg03217995	7:27,203,430	*HOXA9*	3.2	2.1	− 1.4	0.8	0.45
Aortic plaque	cg16913789	7:27,204,005	*HOXA9*	0.8	− 0.2	− 0.1	− 0.2	0.37
Aortic plaque	cg25188395	7:27,204,052	*HOXA9*	0.6	0.8	− 0.2	0.9	0.70
Aortic plaque	cg17466857	7:27,225,528	*HOXA11AS*	0.1	0.0	− 0.1	0.2	0.03
Aortic plaque	cg01419713	8:42,038,135	*PLAT*	0.0	− 1.0	0.8	0.2	0.49
Blood	cg03450842	10:80,834,947	*ZMIZ1*	0.3	0.8	− 0.9	− 0.2	0.26
Blood	cg11660018	11:86,510,915	*OR7E2P*	0.2	0.9	− 0.4	0.3	0.16
Blood	cg03371962	12:1,772,275	*MIR3649*	0.6	0.8	0.1	− 0.5	0.87
Aortic plaque	cg23395715	12:54,369,514	*HOXC11*	0.8	0.6	0.9	0.7	0.68
Aortic plaque	cg02384661	12:54,369,638	*HOXC11*	0.3	0.0	− 0.2	0.3	0.47
Aortic plaque	cg03146625	12:54,448,729	*HOXC4*	1.1	1.9	0.3	1.8	0.42
Aortic plaque	cg15648389	12:54,448,769	*HOXC4*	2.5	0.9	0.9	2.5	0.48
Blood/Carotid plaque	cg05284742	14:93,552,080	*ITPK1*	0.2	1.2	0.2	− 0.2	0.96
Blood	cg17295878	17:77,924,665	*TBC1D16*	0.1	− 0.3	− 0.2	0.1	0.01
Blood	cg03636183	19:17,000,537	*F2RL3*	0.4	0.5	1.0	− 0.5	0.80

Data are shown as the mean change in methylation levels (%) between baseline and 36 months after treatment (calculated as post–pre) for the four groups of women

^a^Chromosome and position (basepair) are based on build 37 (hg19) of the human genome reference sequence

^b^*p*-values are derived from tests of interaction between time-since-menopause and HT using m-values for methylation, with adjustment for age, ethnicity, and estimated blood cell fractions

## Discussion

In the present study, we sought to determine whether changes in methylation levels could represent at least one molecular mechanism for the protective effects of HT on subclinical atherosclerosis as a function of time-since-menopause observed in ELITE [12]. A longitudinal EWAS analysis among a subset of ELITE participants with the lowest and highest 36-month CIMT progression identified two CpG sites on chromosomes 12 and 19 that exhibited highly significant interactions between methylation levels, time-since-menopause, and HT. Specifically, HT resulted in modest, but significant, increases of methylation at both CpGs in the late/HT group but had no effect in the other three groups of women. This was a somewhat surprising finding since we hypothesized that methylation changes in response to HT would be more pronounced in the early/HT group, given that the atheroprotective effects of HT on CIMT progression were only observed among these women [12]. However, we previously observed that estradiol levels were differentially associated with atherosclerosis progression according to timing of HT initiation. For example, CIMT progression rate was decreased with higher estradiol levels among women in early post-menopause but increased among women in the late post-menopause treatment group [23], suggesting potentially adverse effects of HT in this latter group. Alternatively, it is possible that the effects of HT on methylation of leukocytes blood are stronger in women who are 10 or more years since menopause. In this regard, studies have suggested that HT can have certain adverse biological effects in women when initiated in those who are further from menopause [10], which could be reflected at the molecular level by epigenetic modifications in blood cells. Follow-up studies will be required to address this possibility.

The two CpG sites at which methylation levels increased in response to HT in the late post-menopause group were located near *WNT1* (cg19552895) and in *CLEC4M* (cg18515510). While an obvious connection between these genes, HT, atherosclerosis, and methylation changes at either CpG site in blood cells is not presently evident, prior studies have shown that 17β-estradiol can both induce and decrease methylation at CpG sites through *ESR1*-mediated mechanisms [24]. These observations have mostly been in the context of breast cancer and it is not known whether *ESR1*-mediated effects of 17β-estradiol on methylation occurs in blood leukocytes as well. However, the WNT family of signaling molecules have been implicated in various aspects of CVD, including cellular cholesterol homeostasis [25], and circulating levels of WNT1 protein have been reported to be lower in premature myocardial infarction patients than controls during both the acute and stable phases [26]. By comparison, *CLEC4M* is a member of the C-type lectin gene family expressed primarily on endothelial cells in liver and lymph nodes, and plays a role in promoting cellular entry of various viruses [27]. Despite these observations, it is not clear whether the increased methylation observed at cg19552895 and cg18515510 in response to HT would alter expression of *WNT1* and *CLEC4M*, respectively, in blood cells and, more broadly, how the biological function of each protein is directly related to the effects of HT in ELITE participants.

Although strong interactions between HT and time-since-menopause were observed with cg19552895 or cg18515510, we did not obtain evidence for a statistically significant relationship between methylation changes at these CpG sites and changes in free estradiol levels or CIMT progression in the late/HT group. One possible explanation for these observations is that the effects of free estradiol and HT on methylation directly at the level of the vessel wall may not be reflected by epigenetic changes in blood cells. For example, a large EWAS analysis with > 6400 individuals also did not identify any genome-wide significant associations between CIMT and blood cell-derived methylation levels at any CpG site except for cg05575921 at the well-known smoking-associated *AHRR* locus [22]. By comparison, epigenetic analyses with tissue obtained from atherosclerotic and normal carotid, aortic, mammary, or femoral artery samples have identified thousands of associations with atherosclerosis [20–22, 28–30]. Interestingly, several studies observed hypomethylation of CpG sites in atherosclerotic tissue compared to normal arteries and upregulation of multiple pathways that could potentially be causal drivers of plaque development [28, 30]. These findings, taken together with our data, suggest that circulating leukocytes may not be an appropriate surrogate tissue in which to associate methylation modifications with vascular wall phenotypes, even in response to HT. Interestingly, efforts are underway to determine whether methylation profiling of peripheral tissues, such as blood, can provide insight into epigenetic patterns in other tissues [31], which could be applied to epigenetic studies of atherosclerosis-related traits.

While revealing potentially novel epigenetic associations with HT among postmenopausal women, our study should be considered in the context of its limitations. For example, our intention by selecting subsets of women with the lowest CIMT progression from early/HT group, in which the atheroprotective effects of HT were only observed, was to maximize potential molecular differences in response to HT compared to participants from the other three treatment groups. However, this strategy, while efficient, may not have provided sufficient power to detect associations with CIMT due to weak biological effects, particularly since our analyses specifically sought to identify interactions between methylation levels, HT, and time-since-menopause. In addition, although blood cells are the most readily accessible tissue from humans for methylation analyses, they may not, as noted above, adequately reflect atherosclerotic mechanisms at the level of the vessel wall.

## Conclusions

In summary, we evaluated the effects of HT on epigenetic modifications of blood cells in women as a function of time-since-menopause. In addition to replicating previously described associations between smoking and methylation, our study also identified a small number CpG sites at which methylation levels increased in response to HT but only among women who were 10 or more years from menopause. These data represent, to our knowledge, one of the first descriptions of the effects of HT on methylation profiles of blood cells and provide a unique hypothesis-generating dataset that can be leveraged for future studies.

## Methods

### Study population

ELITE was a single-center, randomized, double-blind, placebo-controlled clinical trial (ClinicalTrials.gov number NCT00114517) testing effects of HT on progression of subclinical atherosclerosis as a function of time-since-menopause. Participants were healthy postmenopausal women without diabetes and clinical evidence of CVD who had no menses for at least 6 months or who had surgically induced menopause, as well as a serum estradiol level lower than 25 pg/mL (92 pmol/L). Women in whom time-since-menopause could not be determined, or who had fasting plasma TG levels > 500 mg/dL, diabetes mellitus or fasting serum glucose levels > 140 mg/dL, serum creatinine level > 2.0 mg/dL, uncontrolled hypertension, untreated thyroid disease, life-threatening disease with prognosis < 5 years, a history of deep vein thrombosis, pulmonary embolism, breast cancer, or current use of postmenopausal HT within 1 month of screening were excluded. A total of 643 women were stratified according to time-since-menopause (< 6 years [early] or ≥ 10 years [late]) and randomized to receive either HT or placebo using a 1:1 ratio of stratified blocked randomization, resulting in four treatment groups: early/placebo, early/HT, late/placebo, and late/HT. HT consisted of oral micronized 17β-estradiol 1 mg/day with 4% vaginal micronized progesterone gel 45 mg/day for 10 days each month (among women with intact uterus). ELITE demonstrated that, compared with placebo, HT reduced CIMT progression in women who were within six years of menopause but not women who were 10 or more years from menopause [12]. Additional details on the design, methods, and results of the trial have been described previously [12, 32]. ELITE was approved by the University of Southern California institutional review board and all participants provided written informed consent.

### Whole-genome DNA methylation profiling

To maximize the likelihood of identifying associations between CpG sites and HT as a function of time-since-menopause, we selected subset of 48 women from the early/HT with the lowest rate of CIMT progression over the course of the trial for methylation profiling and an equivalent number of women from each of the early/placebo, late/placebo, and late/HT groups with the highest CIMT progression. Genomic DNA was extracted from buffy coats of this subset of 192 ELITE participants obtained at baseline and 36 months following randomization using DNeasy kits (Qiagen, Valencia, CA) and bisulfite treated with the Zymo EZ DNA Methylation Kit (Zymo Research, Orange, CA). Quantitative levels of DNA methylation were obtained for > 850,000 CpG sites using the Infinium Human Methylation EPIC BeadChip (Illumina, San Diego, CA) according to the manufacturer’s protocols.

### Methylation data processing and normalization

Prior to analysis, the *meffil* package in R [33] was used to carry out several quality control (QC) steps, including filtering of samples and CpG sites, identifying batch effects, and normalizing sample quantiles. *Meffil* is a comprehensive and integrated toolkit that utilizes multiple previously developed R packages for methylation analysis, such as *minfi* [34], *illuminaio* [35], and *noob* [36]. Background and dye-bias correction was first performed using raw probe signal intensities as the input. The *noob* background normalization method [36] was used to account for technical variation in background fluorescence signal, which capitalizes on a new use for the Infinium I design bead types to measure nonspecific fluorescence in the color channel opposite of their design (Cy3/Cy5). Poor quality CpGs were removed using the *illuminaio* R package [35] and the *ChAMP* R package [37–39] was used to identify and exclude SNP-related CpG probes based on previously reported annotations [40]. Low quality samples were removed if they were outliers for methylated/unmethylated levels or control probe means, had too many undetected probes or low bead number probes [33]. Functional normalization (FN) as implemented with the *minfi* R package [34] was used to minimize technical variation based on control probes present in the EPIC BeadChip that do not exhibit biological variation and whose only source of variation is due to technical artifacts. FN was also used to identify the number of principal components (PCs) of methylation matrix to include in the normalization that minimizes the residual variance unexplained by the given number of PCs, and to remove technical artifacts by normalizing sample quantiles using additional fixed and random effects [33]. Quantile normalization was performed using *meffil* where slide, plate, and array were treated as random effects, and the first 10 PCs were included as fixed effects. The Houseman algorithm [41] as implemented in *meffil* was used to estimate fractions of six different white blood cell populations (B cells, CD4 T cells, CD8 T cells, granulocytes, monocytes, and natural killer cells) using GSE35069 as the cell type reference [42]. Leukocyte fraction estimates were subsequently used as covariates in the EWAS analyses. Methylation levels (*β* values) at each CpG site were determined by calculating the ratio of fluorescence intensities between methylated (signal A) and unmethylated (signal B) sites using the formula *β* = Max(M,0)/[Max(M,0) + Max(U,0) + 100]. Thus, *β* values range from 0 (completely unmethylated) to 1 (completely methylated). Prior to final analysis, *β* values were transformed to M-values (log_2_ ratio of methylated vs. unmethylated probe) using ‘beta2m’ function in the *lumi* package in R [43]. The final dataset included 186 ELITE participants in whom methylation data at 748,567 CpG sites were available at both visits (total of 372 methylation profiles at baseline and 36 months after trial randomization).

### Differential methylation analysis

EWAS analyses with smoking were carried out at the baseline and 36-month visits out using linear regression models that were fitted using *limma* [44] as implemented in *meffil* [33]. Participants were categorized as never, former, and current smokers (coded as 0, 1, 2) and methylation M-values were compared across categories using an analysis pipeline in *meffil* with adjustment for age, ethnicity, and estimated blood cell fractions. Longitudinal EWAS analyses with epigenetic data at both the baseline and 36-month visits were used to investigate the effect of HT (treated vs. placebo) and time-since-menopause (< 6 years [early] or ≥ 10 years [late]) on methylation levels. Changes in methylation at CpG sites were tested using *lmrse* package in R [45] designed to fit linear models with cluster robust standard errors across high-dimensional data to evaluate methylation trajectories. Participants for repeated measures analysis were categorized into four groups based on early or late post-menopause and randomized to HT or placebo. P-values for methylation changes at each CpG site were obtained from tests of interaction between these latter four categories and a time variable (baseline vs. 36 months) with adjustment for age, ethnicity, and estimated blood cell fractions.

### Measurement of subclinical atherosclerosis and free estradiol levels

Rate of change in far wall intima–media thickness of the right distal common carotid artery was assessed by computer image processing of B-mode ultrasonograms. At baseline, two examinations were conducted (averaged to obtain baseline CIMT values) and every 6 months during trial follow-up [12]. High-resolution B-mode ultrasonographic imaging and CIMT measurements were performed with use of standardized procedures and in-house technology that was specifically developed for longitudinal measurements of changes in atherosclerosis [32]. Coefficient of variation for baseline CIMT measurements was 0.69% [12]. Plasma estradiol levels were measured at baseline and 36 months after treatment by radioimmunoassay with preceding organic solvent extraction and Celite column partition chromatography, as described previously [23]. The relationship between changes in CIMT progression or free estradiol levels [46] and methylation levels (M-values) at the two CpGs identified through interactions with HT and time-since-menopause was assessed in the late/HT group using partial Spearman’s correlation with adjustment for age and estimated blood cell fractions at baseline and at 36 months after treatment.

## Data Availability

Datasets generated and analyzed during the current study are not publicly available since consent for such public release of epigenetic data from ELITE participants was not obtained. However, raw data to generate figures and tables presented in this study are available from the corresponding author upon reasonable request, with the appropriate permission from ELITE Research Group Committee, and with institutional review board approval.

## Abbreviations

CVD: Cardiovascular disease
HT: Hormone therapy
CIMT: Carotid artery intima-media thickness
ELITE: Early versus Late Intervention Trial with Estradiol trial
EWAS: Epigenome-wide association study
*ESR1*: Estrogen receptor 1
*ESR2*: Estrogen receptor 2
